# Supplementary material for: Identification and functional analysis of non-coding regulatory small RNA FenSr3 in Bacillus amyloliquefaciens LPB-18
Source: PeerJ. 2023 May 15;11:e15236. doi: 10.7717/peerj.15236 (PMC10194069; doi:10.7717/peerj.15236)
Supplement: Supplemental Information 4 [file peerj-11-15236-s004.zip › KO/CK-vs-T1_map/map01212.html]

KEGG PATHWAY: Fatty acid metabolism - Reference pathway


|  |  |
| --- | --- |
| **Fatty acid metabolism - Reference pathway** |  |

[
Pathway menu
| Organism menu
| Pathway entry
|

Hide module list

| User data mapping
|

Image (png) file

]

|  |  |  |
| --- | --- | --- |
| Reference pathway | 184% 150% 122% 100% 82% 67% 55% | 图片下载 |

- **KEGG module**

- Carbohydrate and lipid metabolism
  - Fatty acid metabolism
    - M00082 Fatty acid biosynthesis, initiation- M00083 Fatty acid biosynthesis, elongation
      - M00085 Fatty acid biosynthesis, elongation, mitochondria
      - M00415 Fatty acid biosynthesis, elongation, endoplasmic reticulum
      - M00086 beta-Oxidation, acyl-CoA synthesis
      - M00087 beta-Oxidation

  

- **Reaction module**

- Carboxylic acid metabolism
  - Fatty acid synthesis and degradation
    - RM018 Beta oxidation in acyl-CoA degradation- RM020 Fatty acid synthesis using acetyl-CoA (reversal of RM018)
      - RM021 Fatty acid synthesis using malonyl-CoA
